# Evaluation of Curved Canal Transportation Using the Neoniti Rotary System with Reciprocal Motion: A Comparative Study

**DOI:** 10.1155/2021/4877619

**Published:** 2021-11-24

**Authors:** Mohsen Aminsobhani, Arvin Rezaei Avval, Fatemeh Hamidzadeh

**Affiliations:** ^1^Faculty of Dentistry/Dental Research Center, AJA and Tehran University of Medical Sciences, Tehran, Iran; ^2^Department of Endodontics, Faculty of Dentistry, Tehran University of Medical Science, Tehran, Iran

## Abstract

The ideal root canal preparation is where the original canal morphology is maintained during the biomechanical preparation. Preparation of curved canals has always been a challenge to clinicians. Better results have been suggested for a single NiTi instrument with reciprocating motion than the conventional continuous rotation method in the preparation of curved root canals. Although the Neoniti rotary system is not suggested to be used with reciprocal motion, running a pilot study, we found that it could be possible. The present study aimed to investigate if shaping curved canals using the Neoniti rotary system with reciprocal motion leads to better results in terms of root canal transportation. One hundred acrylic j-shape canal simulator endoblocks were used in this study. Five preparation sequences were applied: GPS followed by A1#20 (GPS + A1#20), GPS followed by A1#20 and then A1#25 (GPS + A1#20 + A1#25), GPS followed by A1#25 (GPS + A1#25), hand file followed by A1#20 (hand file + A1#20), and GPS followed by A1#20 (with reciprocal motion) (GPS + A1#20(reciprocal)). Pictures were taken from blocks once before and once after preparation from two dimensions. Before-and-after pictures were superimposed in Photoshop software. Measurements were performed in Digimizer. The number of autoreverses and pecking motions was recorded after reviewing the recorded videos. Data were analyzed in SPSS, version 26. A *p* value of less than 0.05 was considered statistically significant. The group GPS + A1#20 + A1#25 had more transportation compared with the others, at apical, middle, and coronal thirds not only in the frontal view but also in the lateral view. Other groups were not significantly different. The number of peckings and autoreverses was significantly less when A1#25 was used after GPS and A1#20. When A1#20 was used with reciprocal motion, it had less peckings compared with the same file with continuous rotation, and no autoreverses were observed in that group. Using Neoniti files with reciprocal motion might result in less instrument fatigue and favorable results, with respect to canal anatomy preservation. Using A1#20 before A1#25 also will decrease the stress on the instrument during preparation. However, this may lead to significantly more canal transportation.

## 1. Introduction

Cleaning and shaping of the root canal system is one of the most important phases of root canal treatment which aims to eliminate or at least reduce the intracanal micro-organisms while maintaining the original shape of the root canal [1]. Apical canal transportation is one of the mishaps that changes the shape of the canal and may endanger the treatment outcome [2]. Zipping or perforation of the canal may occur as a result of apical transportation [3]. The conventional use of 2D radiography led to misdiagnosis and underestimation of the real canal curvature. The introduction of 3D modalities of imaging, such as cone beam computed tomography and magnetic resonance imaging, gave a real picture of the root canal system to the clinician before initiation of the treatment [4]. Nickel-titanium (NiTi) rotary systems were introduced into endodontic practice looking forward to less procedural error occurrence, i.e., canal transportation, zip, ledge, and striping perforation [5].

The ideal root canal preparation is where the original canal morphology is maintained during the biomechanical preparation, along with the development of flaring from the coronal to apical portion and preserving the apical foramen [6]. However, the ideal root canal preparation may not be always possible due to the complexity of the root canal anatomy. The biomechanical preparation of the various canal curvatures in the root canals presents great challenge for the clinician in regular endodontic treatment procedures. These curved canals may also limit the ideal mechanical preparation of the root canals and in turn may lead to the development of some procedural errors [5]. Several factors including instrument flexibility, asymmetrical cross-sectional design, and having a radial land have been suggested as key factors contributing to less canal transportation. Instrument flexibility is a multifactorial characteristic which has been proved to be associated with metallurgic properties, taper, size, and cross section [7, 8].

Attempts have been made to reduce the number of files and even to introduce single-file systems, mostly due to infection control concerns [9, 10]. The preparation ability of single-file systems in severely curved root canals has been evaluated in several studies. These systems have been compared with the conventional rotary systems, and results indicated that they could prepare curved canals faster and with less procedural accidents [11, 12].

Considering the motion, single-file rotary systems may be classified as full rotating and reciprocating files. Neoniti A1 (NEOLIX, Châtres-la-Forêt, France) is one of the single-file systems with full rotary motion. This system is made up of special treated alloy leading to a better file flexibility. This system is produced with three different sizes (20/0.06, 25/0.06, and 40/0.04) that are recommended to be used with a speed of 300 to 500 rpm and a torque limit of 1.5 N·cm. According to the manufacturer, this file offers many advantages such as sharp cutting edges, single-file technique, Gothic-like tip design, and built-in abrasive properties [13].

Reciprocal motion consists of a larger counterclockwise rotating angle, which allows the instrument to cut the dentin and a smaller clockwise angle to disengage; due to the greater counterclockwise angle, the instrument continuously progresses toward the apex of the root canal [11, 14, 15]. Better results have been suggested for a single NiTi instrument with reciprocating motion than the conventional continuous rotation method in the preparation of curved root canals. The reciprocal motion is claimed to relieve stress on the instrument, minimize the risk of fracture, and improve cyclic fatigue resistance and lifespan of NiTi instruments [11, 15]. Although the Neoniti rotary system is not suggested to be used with reciprocal motions, running a pilot study, we found that it could be possible.

The present study aimed to investigate if shaping curved canals using the Neoniti rotary system with reciprocal motion leads to better results in terms of root canal transportation at different cross sections.

## 2. Methods and Materials

**Figure 1:**
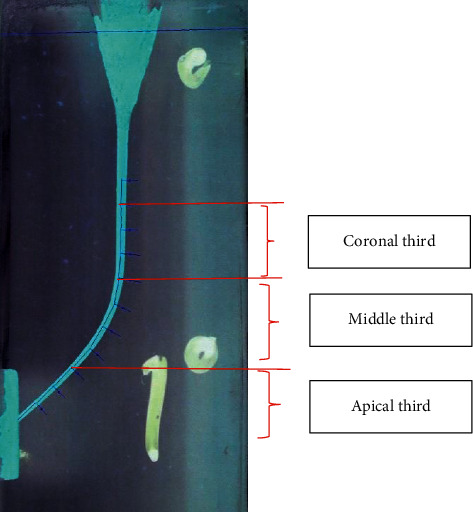
Pattern for transportation measurement of the frontal view of the block.

**Figure 2:**
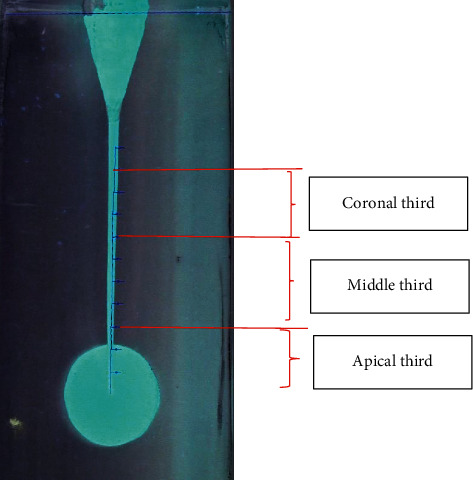
Pattern for transportation measurement of the lateral view of the block.

**Figure 3:**
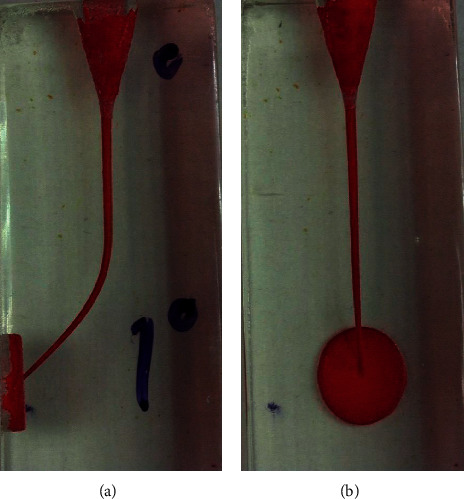
Endoblocks before preparation from the frontal (a) and lateral (b) view.

**Figure 4:**
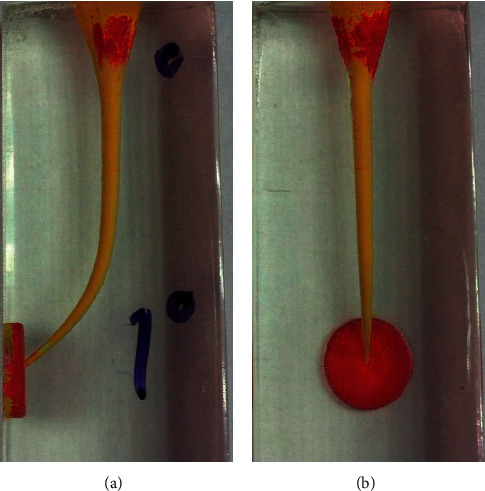
Endoblocks after preparation from the frontal (a) and lateral (b) view.

The present study was conducted on 100 J-shaped acrylic blocks (E-block, Acadental, USA) assigned to 5 groups based on the file sequence used to prepare the simulated J-shape canal: #15 hand file (MANI K-files, MANI, Japan) followed by A1#20, GPS (Neoniti glide path preparation file) followed by A1#20 (continuous rotation), GPS followed by A1#25 (continuous rotation), GPS followed by A1#20 (reciprocal motion), and GPS followed by A1#20 and A1#25 consequently (continuous rotation). Except for the reciprocal motion group, simulated J-shape canals were prepared using the Endo Pilot endorotary motor (Schlumbohm, Brokstedt, Germany) according to manufacturer's catalogue settings, and for better cutting and reducing the number of autoreverses, the torque level was set at 4 N·cm. For the reciprocal motion group, preparation was carried out with a speed of 300 rpm at 2 N·cm torque. Reciprocal motion was manually adjusted as follows: 70 msec. left, 10 msec. pause, and 210 msec. right (i.e., 150° CCW followed by 360° CW in each cycle which was calculated based on slow-motion videos recorded). The canal of blocks was painted with red dye, and a picture was taken from the block once before and once after preparation and painting with yellow dye using a Dino-Lite AM4113 TL stereomicroscope (AnMo Electronics Corporation, New Taipei City, Taiwan). Blocks were put on a white smooth surface, and the photos were taken by using a stereomicroscope from a perpendicular point of view. Each block had two points which were going to be used as constant points to superimpose the taken photos. Before-and-after-preparation pictures were superimposed in Adobe PhotoShop CC 2019 (Adobe Inc., San Jose, California). For both the lateral and frontal view of the block, measurement grid templates were designed to be superimposed on before-and-after-preparation pictures (Figures 1 and 2). Blocks were assessed at 10 cross sections with 1 mm intervals both frontally and laterally in Digimizer image analysis software (MedCalc Software Ltd.) (Figures 3 and 4). Absolute canal transportation at each cross section was calculated as the half of the absolute value of the difference between left- and right-side transportation at that cross section. The mean of the absolute canal transportation at the first, second, and third cross sections was assumed as apical canal transportation. The mean of the absolute canal transportation at the fourth, fifth, sixth, and seventh cross sections was assumed as middle canal transportation. The mean of the absolute canal transportation at the eighth, ninth, and tenth cross sections was assumed as coronal canal transportation. The process of instrumentation was recorded by using a video camera (Sony Corporation, Tokyo, Japan) and rechecked to record the exact number of peckings and autoreverses. Because reciprocal motion has no autoreverse, the number of times hand-piece LED showed red color, i.e., reaching the set torque, was recorded as the number of autoreverses in these groups. To compare the groups' transportation at each of the 3 levels and number of peckings and autoreverses, we used one-way ANOVA combined with Tukey's post hoc statistical tests, using SPSS software ver.26 (IBM Corporation, New York, USA).

## 3. Results

All of the rotary files in the present study were new and used once. Two instrument fractures were observed and excluded: the first one A1#20 was used with continuous rotation after 20 up-and-down motions and 2 autoreverses, and the second one A1#25 was used with continuous rotation after 27 up-and-down motions and 6 autoreverses.

### 3.1. The Frontal View

In the apical third of the canal, the transportation comparison was as follows: the group GPS + A1#20 + A1#25 had more transportation compared with the others; however, this difference with the group GPS + A1#25 was not statistically significant. The latter had no statistically significant difference with other groups. None of the other groups showed a statistically significant difference. In the middle and coronal third of the canal, the transportation comparison was as follows: the group GPS + A1#20 + A1#25 had statistically significant more transportation compared with the others (*p* value < 0.05). No statistically significant difference was observed between other groups (Table 1).

### 3.2. The Lateral View

In the apical and middle thirds, the group GPS + A1#20 + A1#25 had statistically significant more transportation compared with the others. In the coronal third, the group GPS + A1#20 + A1#25 had more transportation compared with the others, and these differences were statistically significant (*p* value < 0.05) except for the group hand file + A1#20. Other groups did not have any significant differences (Table 2).

### 3.3. Number of Peckings and Autoreverses

The data suggest that when a glide path has already been prepared, using A1#25 after A1#20 would lead to significantly less pecking motions and autoreverses compared with using either A1#20 or A1#25 separately (*p* value < 0.001). When A1#25 is used immediately after GPS, significantly more pecking motions and autoreverses than other groups during shaping were observed (*p* value < 0.001). When A1#20 is used with reciprocal motion, less but not statistically significant pecking motions compared with A1#20 with continuous rotation were observed. Using A1#20 with reciprocal motion was associated with significantly less autoreverses compared with other files (*p* value < 0.001). According to Table 3, this group had no autoreverse; i.e., during instrumentation in this group, torque never met the set torque. When hand file is used instead of GPS to prepare the glide path, A1#20 would have less autoreverses during instrumentation (*p* value = 0.001) (Tables 3 and 4).

## 4. Discussion

This study was conducted on 100 j-shape canal simulant endoblocks to investigate the canal transportation of the Neoniti rotary system when it is, contradictory to the manufacturer's suggestions, used with reciprocal motion. Apical transportation in curved canals is important because the curvature of the canal affects the access for instrumentation and increases the risk of fracture of an endodontic file inside the canal [16]. The apical transportation is defined as the elimination of the dental structure in the outer part of the curvature of the apical third of the root canal [17]. This is due to the tendency of the endodontic files to recover their original shape during the instrumentation of the root canals, and this could lead to the creation of a zip and a possible perforation. Considering the better results of using NiTi files with reciprocal motion, reported by previous studies [11, 15], we conducted the present one. The aim of the present study was to investigate if there is any difference between using the Neoniti system as a single-file system and as a multiple-file system and also if there is any difference between using this system with a continuously rotating motion and reciprocal motion.

For continuously rotating motion groups, preparation was carried out with a speed of 300 rpm at 4 N·cm torque. This torque is larger than that in the manufacturer's instruction. It was because of the hardness of the acrylic blocks. In other words, the instrument was not able to cut the walls of the canal with lower torque levels without high number of autoreverses. The torque level for reciprocal motion was set to half of the full rotation motion.

Instrument fracture, as a great challenge for clinicians, has been mainly attributed to cyclic fatigue and torsional resistance of the instrument. These factors have been investigated in several studies and are proved to be related to metallurgic properties [18], metal mass, cross-section area, shaft length, and a more important but more recently investigated factor, i.e., polar moment of inertia [19, 20]. Neoniti has been formerly studied and proved to have favorable results concerning fatigue resistance [21]. Based on the data of the number of pecking and autoreverses, reciprocal motion led to less pecking and less autoreverses compared to continuous rotation although the torque was lower. It means that, by using the same rotary system with reciprocal motion instead of continuous rotation, the stress on the file during the process of shaping is reduced. As it has been mentioned in previous studies by Glassman et al., De-Deus et al., You et al., and Gambarini et al. [22–25], reciprocating motion can extend cyclic fatigue resistance of NiTi instruments when compared to continuous rotation. In other words, it reduces the risk of instrument fracture as well.

Based on the data of the mean transportation in each group, results indicate that although the size of the last instrument used for shaping is a determinant of transportation, the duration of instrumentation is more important, which is why the group GPS + A1#20 + A1#25 had significantly more transportation at each level either in the frontal or lateral view.

Similar to the previous study by Zhao et al. [26], there were no statistically significant difference between reciprocal motion (Wave One) and continuous rotation (ProTaper Next, ProTaper Universal) in middle and coronal third of the canal. They reported less canal transportation in the group with continuous rotation which may be due to the different apical taper of the files used in the group with continuous rotation. The present study showed that apical transportation is not different when files are used neither with reciprocal motion nor with continuous rotation. There are studies by Berutti et al. [27] and Yoo and Cho [28] which concluded that Wave One leads to less transportation, and their results are in contrast with the results of recent studies by Giuliani et al. and Marzouk et al. [29, 30]. It must be noted that, in these studies, Wave One had been used with a pecking motion instead of brushing motion, which might be a confounding factor in the studies using two different rotary systems.

The baseline diameter of the canal in blocks was 0.2 mm. It is suggested to enlarge the canals up to #20 hand file before using rotary instruments [31]. That is why we used these blocks. Keeping in mind that mostly the canal diameter is much less, it would be concluded that, in proportion to real canal dimensions, the amount of transportation might be more than observed.

In contrast with the frontal view, in the lateral view, transportation in the middle third is more than in the coronal third. It might be due to the file's tendency to straighten the curve of the canal and also the special direction of the curve in one dimension, which may deviate the file during instrumentation.

The present study tried to compare the transportation produced by the Neoniti rotary system when used in continuous rotation vs. reciprocal motion in resin blocks. Because the transportation produced by these files on dentin might be quite different from that on resin blocks, it is suggested for future studies to assess whether the same results would be expected in the extracted teeth and clinically.

## 5. Conclusions

Using Neoniti files with reciprocal motion might result in less instrument fatigue and favorable results, with respect to canal anatomy preservation. Using A1#20 before A1#25 also will decrease the stress on the instrument during preparation. However, this may lead to significantly more canal transportation. The single-file technique is recommended for this system.

## Figures and Tables

**Table 1:** Transportation in the frontal view (mm).

Group	Mean apical third transportation (mm)	Mean middle third transportation (mm)	Mean coronal third transportation (mm)
GPS + A1#20	0.025 ± 0.012	0.042 ± 0.015	0.124 ± 0.069
GPS + A1#20 + A1#25	0.045 ± 0.025	0.131 ± 0.052	0.185 ± 0.082
GPS + A1#25	0.029 ± 0.022	0.056 ± 0.024	0.115 ± 0.080
Hand file + A1#20	0.019 ± 0.009	0.063 ± 0.023	0.089 ± 0.064
GPS + A1#20 (reciprocal motion)	0.028 ± 0.019	0.060 ± 0.023	0.087 ± 0.050

**Table 2:** Transportation in the lateral view (mm).

Group	Mean apical third transportation (mm)	Mean middle third transportation (mm)	Mean coronal third transportation (mm)
GPS + A1#20	0.016 ± 0.094	0.061 ± 0.026	0.055 ± 0.035
GPS + A1#20 + A1#25	0.050 ± 0.023	0.115 ± 0.062	0.101 ± 0.050
GPS + A1#25	0.027 ± 0.018	0.069 ± 0.047	0.054 ± 0.046
Hand file + A1#20	0.022 ± 0.012	0.069 ± 0.038	0.064 ± 0.044
GPS + A1#20 (reciprocal motion)	0.021 ± 0.012	0.060 ± 0.044	0.058 ± 0.050

**Table 3:** Number of autoreverses (GPS is in a single row because it was used in different groups).

Group	File	Mean	Std. deviation	Minimum	Maximum
	GPS	0.03	0.26	0	2
GPS + A1#20	A1#20	9.75	1.94	6	14
GPS + A1#20 + A1#25	A1#20	9.75	1.94	6	14
	A1#25	4.65	2.62	2	13
GPS + A1#25	A1#25	19.25	4.29	7	26
Hand file + A1#20	Hand file				
	A1#20	7.20	1.61	4	10
GPS + A1#20 (reciprocal motion)	A1#20	0	0	0	0

**Table 4:** Number of peckings (GPS is in a single row because it was used in different groups).

Group	File	Mean	Std. deviation	Minimum	Maximum
	GPS	22.95	6.54	5	39
GPS + A1#20	A1#20	36.50	3.79	30	44
GPS + A1#20 + A1#25	A1#20	36.50	3.79	30	44
	A1#25	14.20	4.14	8	25
GPS + A1#25	A1#25	48.90	8.09	28	62
Hand file + A1#20	Hand file				
	A1#20	32.95	7.34	22	48
	A1#20	34.10	5.14	24	47

## Data Availability

The data used to support the findings of this study are available from corresponding author upon request.
